# Sodium–glucose transporter-2 inhibitors for prevention and treatment of cardiorenal complications of type 2 diabetes

**DOI:** 10.1186/s12933-021-01213-w

**Published:** 2021-01-11

**Authors:** Dario Giugliano, Miriam Longo, Lorenzo Scappaticcio, Paola Caruso, Katherine Esposito

**Affiliations:** 1 Division of Endocrinology and Metabolic Diseases, Department of Advanced Medical and Surgical Sciences, University of Campania Luigi Vanvitelli, Naples, Italy; 2 Ph.D. of Translational Medicine, Chair of Endocrinology and Metabolic Diseases, Department of Advanced Medical and Surgical Sciences, University of Campania Luigi Vanvitelli, Naples, Italy; 3 Diabetes Unit, Department of Advanced Medical and Surgical Sciences, University of Campania Luigi Vanvitelli, Naples, Italy

**Keywords:** SGLT-2 inhibitors, Type 2 diabetes, MACE, Heart failure, Diabetic kidney disease, Age, Primary and secondary prevention, Class effect

## Abstract

Hospitalization for major diabetes complications, including myocardial infarction, stroke, lower-extremity amputation, and end-stage kidney disease, is on the rise and represents a great health burden for patients with type 2 diabetes (T2D), in particular for older people. Newer glucose-lowering medications have generated some optimism on the possibility to influence the natural history of cardiorenal complications of T2D. This review summarizes work in the area of sodium–glucose cotransporter 2 inhibitors (SGLT-2i) treatment and prevention of cardiorenal complications in patients with T2D (major adverse cardiovascular events, hospitalization for heart failure, kidney outcomes), with a particular emphasis on the effect of age, the role of primary versus secondary prevention and the possible extension of their cardiorenal benefits to the entire class of SGLT-2i.

## Introduction

In the first half of the past decade, hospitalization for major diabetes complications, including myocardial infarction (MI), stroke, lower-extremity amputation, and end-stage kidney disease (ESKD), has increased substantially, subtracting much of the benefits obtained during the years between 1990 and 2010 [1]. The future of diabetes care may be darkened by this apparent resurgence of vascular complications, as well as by the evidence that heart failure (HF), once a neglected complication, may be as common as coronary heart disease in patients with T2D [2, 3]. Accordingly, there has been a paradoxical increase of 11% in the rates of HF in young adults with diabetes participating in the National Health Interview Survey 1985–2014 [4]. These US data are consistent with both Swedish [5] and UK [6] data indicating that patients with T2D who obtained the control of five variables to optimal values (hemoglobin A1c [A1C], total cholesterol or LDL-cholesterol, blood pressure, absence of albuminuria or triglycerides, and abstinence from smoking) were at higher risk (31 to 45% higher risk) of hospitalization for HF. The natural history of HF in T2D seems to be unaffected by the optimal control of major metabolic and cardiovascular (CV) risk factors.

Life expectancy in the U.S. has declined since 2014 [7], which has been attributed, in part, to increased prevalence of underlying cardiometabolic risk factors, including obesity and diabetes. Moreover, the estimated number of people over 65 years of age with diabetes was 111 million in 2019 and will reach 276 million by 2045 [8]. Older people with T2D are especially vulnerable to cardiovascular disease (CVD) which remains the leading cause of death globally [9]. Consequently, prevention and optimal treatment of CVD in this population will be a major worldwide health policy challenge during the next decades.

## Glycemic control is not enough

Only 9% of the risk of MACE (major adverse cardiovascular events) is eliminated after achievement of the best possible glycemic control in patients with T2D and the risk of HF is not influenced at all [2]. This evidence has generated the concept of residual vascular risk, i.e. the risk of vascular event that persists high to very high after intensive glucose control despite the attainment of near‐to‐normal A1C targets [10, 11]. Moreover, among adults with T2D and pre-existing CVD, there is no difference in the risk of CV events in those allocated to intensive glucose control compared with those in the standard care arm (hazard ratio = 0.98, 95% confidence interval, 0.87–1.09) [12]. Paradoxically, tight glycemic control is ineffective on CV risk in those T2D patients who are at their maximum risk for future CV events. Finally, tight glycemic control, especially in older patients with multiple medical conditions, is associated with an increased risk of hypoglycemia [13, 14], which may further increase CV risk [15].

The evidence so far accumulated seems in contrast with the old paradigm that therapeutic efforts for patients with T2D should be devoted to the control of hyperglycemia for preventing the development of micro- and macrovascular complications. However, the contrast can only be apparent, as the CV risk in T2D can only be cleared off by the simultaneous control of the multiple factors at play, including glucose, lipids, blood pressure, albuminuria, and smoking. As detailed before, HF is a pathological condition that is not affected by the control of major CV risk factors. Furthermore, chronic kidney disease (CKD) has an independent role in dictating the CV prognosis of patients with T2D [16]. The cornerstone of therapy to prevent diabetic kidney disease (DKD) is the strict control of blood pressure with the renin–angiotensin–aldosterone system blockade and blood glucose levels [17]. However, many patients with T2D progress to DKD despite standard treatment. There is an unmet clinical need to prevent or delay DKD progression; as a logical consequence, the optimal anti-hyperglycemic drug should be the one that also improves the cardiorenal outlook of patients with T2D.

## SGLT-2 inhibitors

Sodium-glucose cotransporter 2 inhibitors (SGLT-2i) are a new class of orally active drugs approved for the management of T2D [18]. They are also known as gliflozins, by the founder phlorizin which was shown to cause glycosuria in 1886. However, inadequate pharmacokinetic characteristics, such as low oral availability and short half-life, hampered phlorizin use as a therapeutic agent. Dapagliflozin, canagliflozin, empagliflozin and ertugliflozin are the SGLT-2i that have been investigated in cardiovascular outcomes trials (CVOTS) and are available in both US and EU. Other SGLT-2i are ipragliflozin, luseogliflozin, and tofogliflozin, all launched in Japan, sotagliflozin, approved in UE for certain patients with type 1 diabetes, and remogliflozin approved in India [19].

SGLT-2i have a unique mechanism of action, as they mediate the reabsorption of about 90% of the filtered glucose (approximately 200 g), because the overall process has a high capacity [17]. As a consequence, inhibition of glucose reabsorption at the level of the proximal tubule results in enhanced glycosuria, osmotic diuresis and natriuresis, thereby improving glucose control, with a limited risk of hypoglycemia. Moreover, SGLT-2i exert additional positive effects on overall CV risk by lowering body weight and blood pressure. They are oral drugs, which may also fit with the exhortation by the American Diabetes Association (ADA) to limit complex therapies, especially for older adults with diabetes [20]. Simplification of therapy at every level could contribute to increase the adherence to antidiabetic therapy, which may range from 38.5 to 93.1% [21]. For instance, Gordon et al. [22] used the real-world data from 33,849 patients with T2D in UK and found that antihyperglycemic drugs associated with weight loss and lower incidence of hypoglycemia were generally associated with better medication adherence, which in turn was related to improved glycemic control. Medication non-adherence may also contribute for up to 75% to the gap between clinical efficacy from randomized controlled trials and real world results in lowering A1C levels [23, 24].

## Cardiorenal effects

### Cardiovascular events

**Fig. 1:**
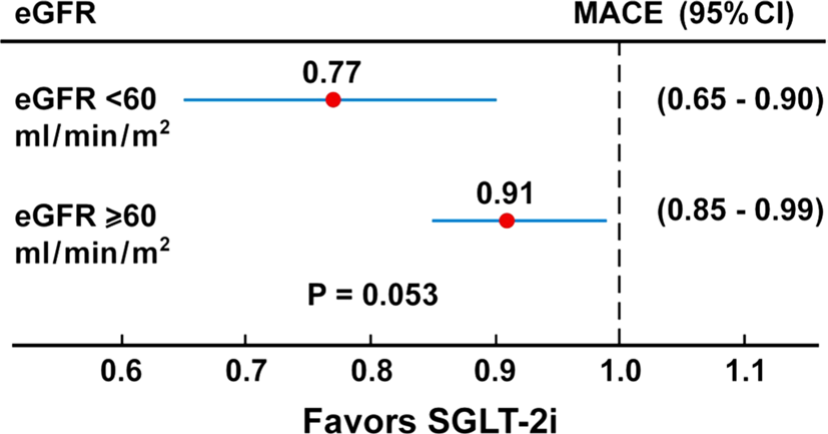
Meta-analysis evaluating the efficacy of SGLT-2i (empagliflozin, canagliflozin and dapagliflozin) on MACE according to basal eGFR values in four trials (EMPA-REG, CANVAS, DECLARE and CREDENCE)

Overall, CVD affects approximately about 32% of all persons with T2D [25] in whom CV risk is mitigated primarily with statins and aspirin [26]. Recent meta-analyses of CVOTs with empagliflozin, canagliflozin or dapagliflozin reported a statistically significant 11–12% reduction in MACE [27, 28], with benefit only seen in patients with atherosclerotic cardiovascular disease (14% reduction) and not in those without (0% reduction, P for interaction = 0.0501) [27]. Most patients assessed in the trials (EMPA-REG [29], CANVAS [30], DECLARE [31], CREDENCE [32]) had preserved kidney function (estimated glomerular filtration rate [eGFR] ≥ 60 ml/min/1.73 m^2^), with 7754 participants (20%) presenting a baseline eGFR < 60 ml/min/1.73 m^2^. Patients with reduced kidney function achieved greater proportional risk reductions for MACE (23% reduction) than patients with preserved kidney function (9% reduction), although the difference was of borderline significance (P for heterogeneity between subgroups = 0.053) (Fig. 1) [33].

The data suggest that the CV benefits of SGLT-2i may even be greater in patients with T2D and DKD. This interpretation has recently found a support from the results of the SCORED trial [34] which randomized 10,584 patients with T2D and DKD (eGFR values between 25 and 60 ml/min/1.73 m^2^), with or without albuminuria, to sotagliflozin or placebo: compared to placebo, sotagliflozin resulted in a lower risk of MACE (16%, P = 0.035), associated with a robust and significant reduction of 32% total MI and 34% total stroke. Sotagliflozin not only inhibits SGLT-2 but also SGTL-1, which primarily resides in the gastrointestinal tract and is the main route for gut absorption of glucose. The activity of sotagliflozin against the SGLT-1 protein likely explains its ability to cut A1C levels in patients with severe renal dysfunction, a condition that opposes glucose lowering by SGLT-2i.

### Heart failure

Hospitalization for HF was significantly reduced by 31–32% with SGLT-2i [27, 28], whether the meta-analyses included three (EMPA-REG, CANVAS, DECLARE) or four (+CREDENCE) trials. Since then, other outcome trials have been published: specifically, the VERTIS-CV trial [35] with ertugliflozin (8246 patients with T2D randomized to ertugliflozin or placebo and followed for a mean of 3.5 years), the DAPA-HF trial [36, 37] with dapagliflozin (4744 patients with or without T2D and with HF randomized to dapagliflozin or placebo and followed for a median of 18.2 months), the EMPEROR-Reduced trial [38] with empagliflozin (3730 patients with or without T2D and with HF randomized to empagliflozin or placebo and followed for a median of 16 months), the SCORED trial [34] with sotagliflozin (10 584 patients with T2D and DKD randomized to sotagliflozin or placebo and followed for 16 months) and the SOLOIST-WHF trials [39] with sotagliflozin (1222 patients with T2D recently hospitalized for worsening HF randomized to sotagliflozin or placebo and followed for 9 months). The primary outcome varied across trials: MACE (EMPA-REG, CANVAS, DECLARE, VERTIS-CV), composite of end-stage kidney disease, doubling of the serum creatinine level, or death from renal or cardiovascular causes (CREDENCE), composite of worsening heart failure or cardiovascular death (DAPA-HF, EMPEROR-Reduced), deaths from cardiovascular causes, hospitalizations for heart failure, and urgent visits for heart failure (SCORED, SOLOIST-WHF).

**Fig. 2:**
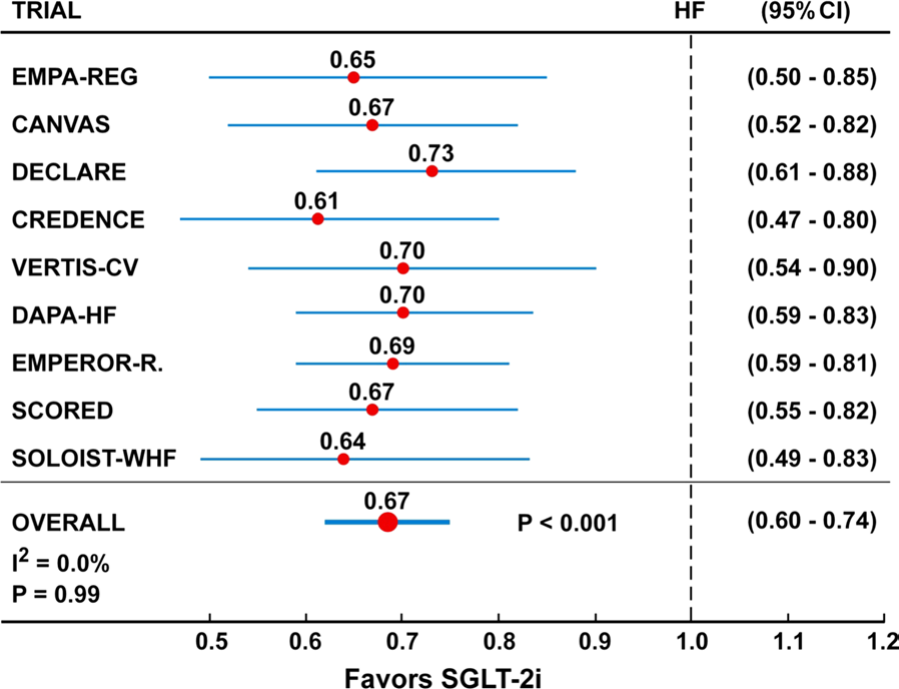
Random effect meta-analysis of nine trials with five SGLT-21 (empagliflozin, canagliflozin, dapagliflozin, eurtugliflozin, sotagliflozin) on hospitalization for heart failure (HF) in patients with or without T2D, and with or without HF at baseline. Overall hazard ratio (HF) is 0.67 (95% Confidence Intervals 0.60–0.74)

We did a random effect meta-analysis including all the nine trials that evaluated the effect of the five SGLT-2i (empagliflozin, canagliflozin, dapagliflozin, ertugliflozin, sotagliflozin) in 67,190 patients with or without T2D, and with or without HF at baseline, on hospitalization for HF. Pooled summary estimates were calculated according to the random effects model, using the empirical Bayes method [40]. The overall effect showed a 32% reduction of the risk of hospitalization for HF in those taking the SGLT-2i than those taking placebo (Fig. 2). The absence of heterogeneity and the strict range of 95% confidence intervals allow to predict an estimate of the true mean of HR likely to be within 0.60 and 0.74, indicating a reduction of the risk ranging from 40 to 26%.

An indirect support for this estimate comes from the results of the DAPA-CKD trial [41] evaluating 4304 patients with or without T2D, and with CKD randomized to dapagliflozin or placebo and followed for a median period of 2.4 years: the hazard ratio for the composite endpoint of CV death and hospitalization for HF was 0.71 (95% CI 0.55–0.92), but that for HF alone, although not presented in the results, should even be lower, as the HR for CV death alone was 0.81 (0.58–1.12).

The results of the present meta-analysis gives further support for the use of SGLT-2i to provide symptomatic improvement in HF symptoms and hospital presentations, as well as deaths, in subjects both with and without T2D [42]. The benefits of SGLT-2i on HF occur early after randomization, are independent of the glycemic status of patients and unrelated to glycemic control in patients with T2D [43]. Moreover, both SCORED [34] and SOLOIST-WHF [39] trials included a percentage of diabetic patients with HF and preserved ejection fraction (left ventricular ejection fraction of at least 50%); both trials found that sotagliflozin was effective in reducing hospitalization for HF, which may remove some hesitation to use SGLT-2i in T2D patients with HF and preserved ejection fraction. Moreover, SOLOIST-WHF was the first trial that evaluated patients with T2D recently hospitalized for worsening HF, although in stable conditions.

The combination of β-blockers, renin angiotensin system blockers with neprilysin inhibitors, and mineralocorticoid receptor antagonists reduces mortality among patients with HF and reduced ejection fraction by an estimated 63% vs placebo, to which it can be added a further 2.3% absolute risk reduction in all-cause mortality at 18 months from SGLT-2i. A combination of these four medicines represents one of the most cost-effective intervention in reducing the risk of death in patients with HF [44]. For instance, it has been calculated that about 80% of patients with HF and reduced ejection fraction would be potentially eligible for initiation of dapagliflozin based on the US Food and Drug Administration label, and independent of the presence of T2D [45].

### Kidney disease

Up to 40% of individuals of the US adult diabetic population have some form of kidney disease [32]. In 4 outcome trials (EMPA-REG, CANVAS, DECLARE and CREDENCE), SGLT-2i reduced by 33% the risk of dialysis, transplantation or death due to kidney disease, reduced by 35% ESKD and by 25% acute kidney injury. The benefits were consistent across studies and for all eGFR subgroups, including participants with a baseline eGFR 30–45 ml/min/1.73 m^2^ [46]. Moreover, in the DAPA-CKD trial [41], which assessed the effect of dapagliflozin in patients with CKD, with or without T2D, the primary outcome (a composite of a sustained decline in the eGFR of at least 50%, ESKD, or death from renal or CV causes) was reduced by 39% (HR = 0.61; 95% CI 0.51 to 0.72, P < 0.001).

At the present, SGLT2-i, at least empagliflozin, canagliflozin and dapagliflozin, are the glucose-lowering agents that are associated with the most impressive overall renal protection, with a positive combined effect on albuminuria, eGFR decline and progression to ESKD within the context of renin–angiotensin–aldosterone system (RAAS) blockade therapy. Treatment with these SGLT-2i could be useful for early [47] as well as late [48] prevention of DKD. On the other hand, both VERTIS-CV trial [35] with ertugliflozin and SCORED trial [34] with sotagliflozin failed to show any significant effect on kidney endpoints, casting some doubt about a class effect of SGLT-2i for renal protection.

### Proposed mechanisms

Several hypotheses have been postulated to explain the benefit on cardiorenal outcomes observed with SLGT-2i. The fuel hypothesis [49] postulates that SGLT-2i cause a mild but persistent increase in the production of ketone bodies, in particular beta-hydroxybutyrate, which becomes, along with free fatty acids, the main substrates for ATP production in the myocardium, in detriment of glucose. The sodium-hydrogen exchanger hypothesis [50] claims that SLGT-2i may directly bind to and inhibit the sodium-hydrogen exchangers in the heart and kidney. The smart diuretic hypothesis [51] suggests that the cardiorenal benefits observed with SGLT-2i are in part due to their more selective diuretic effects, leading to osmotic diuresis. The tubule-centered hypothesis [52] asserts that tubular growth is associated with the development of a senescence-like molecular signature that sets the stage for inflammation and fibrosis; by attenuating the proximal reabsorption of sodium and glucose, SGLT-2i mitigate hyperfiltration and normalize tubule-glomerular feedback signals. Moreover, the list of their cardiorenal protective effects is expanding and includes, among others, preventing adverse cardiac remodeling and ischemia/reperfusion injury, increasing circulating hematopoietic progenitor cells, decreasing epicardial fat mass, decreasing oxidative stress and inflammation, increasing erythropoietin levels and inhibiting the sympathetic nervous system [53, 54].

Attractive these hypotheses may be, they must compare with the results of mechanistic studies. For example, dapagliflozin did not produce a meaningful decline in natriuretic peptides in a smaller mechanistic study in patients with HF with reduced ejection fraction [55], suggesting that enhanced diuresis and decongestion may not be a primary driver of the reduced risk of outcomes seen in DAPA-HF trial [36]. Further research will be needed to identify the mechanisms of action of dapagliflozin and other SGLT-2i in patients with HF.

## The effect of age

Nearly 50% of patients with T2D are 65 years old or older [8]. In older patients, treatment with SGLT-2i is effective at lowering glucose, body weight and systolic blood pressure, as shown in a meta-analysis of post-hoc studies in which the median age of patients was > 60 years [56]. On the other hand, specific trials that evaluated both the efficacy and safety of SGLT-2i in older patients with T2D, especially in those > 75 years, are still lacking and only very limited data are available from observational studies [57]. The post-hoc analysis of both EMPA-REG [58] and DECLARE [59] trials confirms that the efficacy/safety profile of these two SGLT-2i is unchanged by age, even with some signal of greater efficacy in patients older than 65 years of age. In particular, a significant interaction (P = 0.01) was reported in EMPA-REG trial [58] regarding the primary cardiovascular composite end point with better results in patients with T2D aged ≥ 65 years versus < 65 years.

**Fig. 3:**
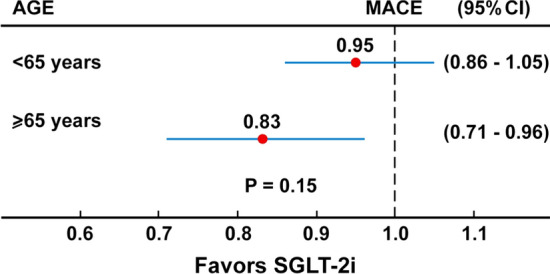
Meta-analysis evaluating the efficacy of SGLT-2i on MACE according to the age at entry in three trials (EMPA-REG, CANVAS and DECLARE)

Figure 3 shows the results of a meta-analysis [60] on the effects of three SGLT-2i of the risk of MACE stratified by age (< 65 years vs ≥ 65 years). The percentage of diabetic patients ≥ 65 years ranged from 44.5% (EMPA-REG), to 46% (DECLARE), to 60% (CANVAS). The hazard ratio for MACE was 0.95 in people < 65 years and 0.83 for people ≥ 65 years, and the effect on MACE was only significant in people of 65 years of age or older. However, the absence of interaction between subgroups (P = 0.15) suggests that the effect of intervention on MACE was similar in participants of both age groups.

In the SCORED trial [34] with sotagliflozin, the primary efficacy endpoint of total occurrences of cardiovascular deaths, HF hospitalizations, and urgent visits for HF was not significantly different in the 3224 (30.5%) patients aged < 65 years (HR = 0.60, 95% CI 0.43–0.83) vs the 7360 (69.5%) patients aged ≥ 65 years (HR = 0.79, 0.66–0.95). Furthermore, according to a post- hoc analysis of the data of DAPA-HF [61], dapagliflozin reduced the risk of death and worsening HF and improved symptoms across a broad spectrum of age (< 55 to ≥ 75 years), without safety concerns even in older individuals. Similarly, in the DAPA-CKD trial [41], the reduction of the primary endpoint (a composite of a sustained decline in the eGFR of ≥ 50%, ESKD, or death from renal or cardiovascular causes) with dapagliflozin was of the same magnitude in both patients aged < 65 and ≥ 65 years. Overall, the results are reassuring, as they confirm that the efficacy profile of SGLT-2i is unchanged by age.

## Class effect of SGLT-2i

The definition of class effect for a drug may be based on three concepts: a similar chemical structure, a similar mechanism of action and similar pharmacological effects [62]. SGLT-2i are considered a class as they share a similar chemical structure derived from the ancestor phlorizin, a similar mechanism of action related to the inhibition of SGLT-2 located on the luminal membrane of proximal tubular cells (brush border), and similar pharmacological effects at the level of kidney, resulting in glycosuria and osmotic diuresis. In order to make the concept of a class more stringent, we have assumed that a class effect is paramount when an effect on a particular outcome is present and is significant for each drug within the class of SGLT-2i [19].

**Fig. 4:**
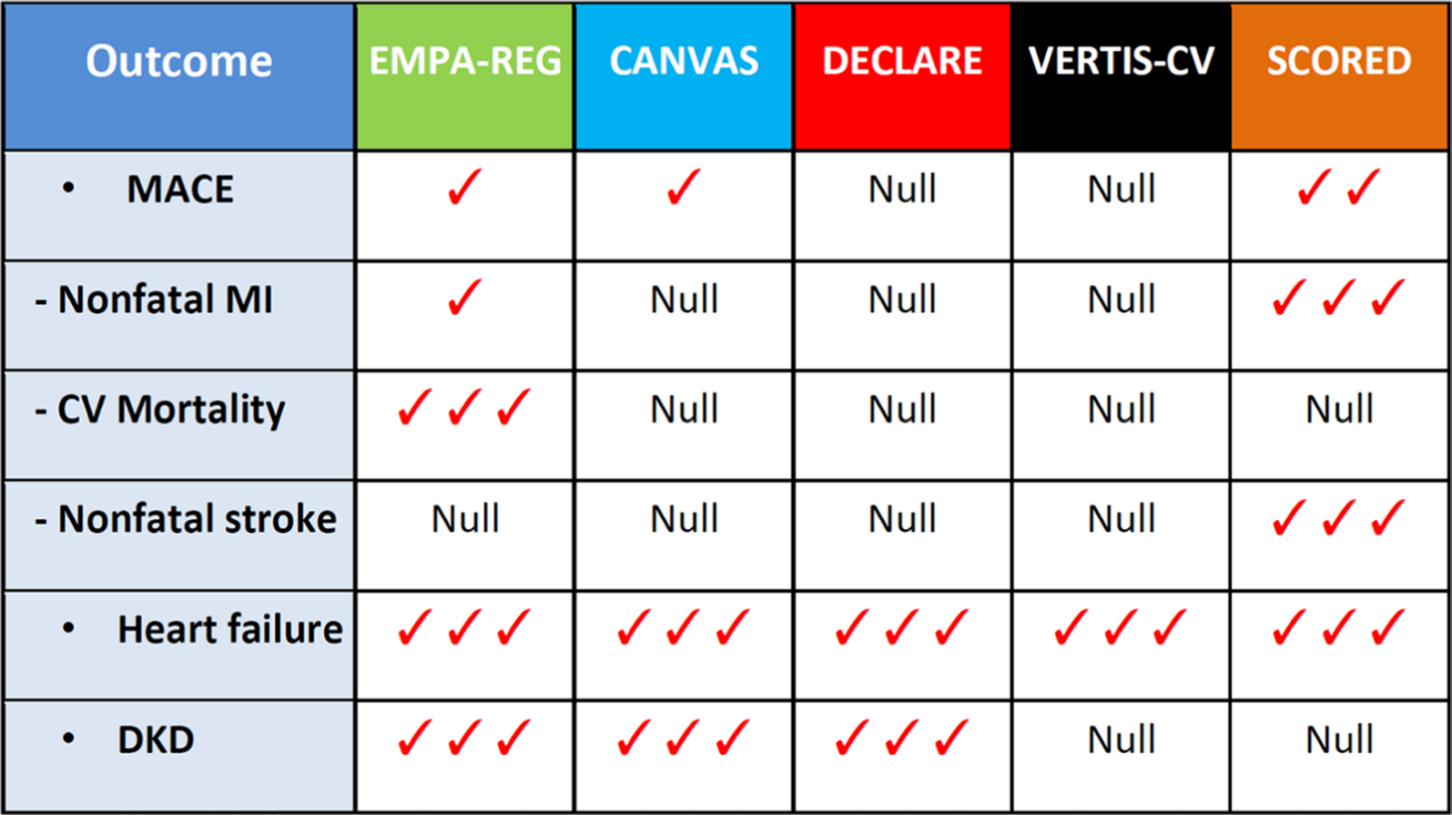
Scenario of cardiorenal effects SGLT-2i in patients with T2D participating in CVOTs. MACE, major adverse cardiovascular events; DKD, diabetic kidney disease.  : modest benefit (the hazard ratio -HR- for a given endpoint during treatment with a gliflozin is > 0.85 and < 1.0, as compared with placebo).  : moderate benefit (HR ≤ 0.85 and > 0.75).  : large benefit (HR ≤ 0.75)

Figure 4 shows the effects of the five SGLT-2i (empagliflozin, canagliflozin, dapagliflozin, ertugliflozin and sotagliflozin) tested in outcome trials according to the more stringent definition of class effect, as detailed above. The outcomes considered include MACE (with its three components of nonfatal MI, CV mortality and nonfatal stroke), which was the primary outcome in EMPA-REG [29], CANVAS [30], DECLARE [31], VERTIS-CV [35] and SCORED [34], hospitalization for HF and progression of DKD, which were secondary outcomes. Because of the early closing of the SCORED trial and the fewer than planned number of events, the primary end point was changed during the trial to the composite of the total number of deaths from CV causes, hospitalizations and urgent visits for HF.

For MACE, there seems to be no class effect, as both DECLARE and VERTIS-CV failed to reduce it significantly: on the other hand, a class effect is evident for hospitalization for HF, which at present remains the more striking therapeutic effect of SGLT-2i. Unfortunately, both VERTIS-CV and SCORED trials were unable to show any significant effect on DKD progression, a renal composite outcome of death from renal causes, kidney replacement therapy, or doubling of the serum creatinine level. Although comparison between trials should always be done with caution, the five trials are generally consistent with each other, showing reliable cardiorenal benefits (with the exception of VERTIS-CV and SCORED for DKD progression) and comparable expected adverse effects.

## Primary vs secondary prevention

The definition of secondary prevention usually refers to patients who suffered a previous acute ischemic event, whereas all other patients are categorized as requiring primary prevention. The distinction between primary and secondary prevention risk categories in diabetes is no longer reported in European Society of Cardiology guidelines on diabetes, pre-diabetes, and CVD developed in collaboration with the European Association for the Study of Diabetes [63]. Accordingly, the CV risk in patients with T2D may range from the highest risk in patients who experienced a previous CV event, to a mild risk in patients with the main risk factors within target ranges, a very small category of about 5%-6% of patients [5, 6, 63, 64]. The CVOTs used to distinguish T2D patients with established CVD (secondary prevention), that is, those having experienced a previous event or with evidence of more than 50% stenosis in an epicardial coronary artery, and patients without established CVD (primary prevention), i.e. those with many risk factors for CVD. However, definitions of established CVD varied across trials; although most patients had experienced a previous event at the level of coronary, cerebral or peripheral arteries, patients without events, but with clinical or instrumental evidence of the presence of CVD, were also included.

Data from 57 articles including a population of 4,549,481 persons with T2D suggest a prevalence of CVD of 32.2%, i.e. about one third of T2D population may have established CV disease [24]; however, this epidemiological figure is overturned in CVOTs, in which the percentage of participating with established CVD at baseline was 65% (Table 1). In order to ensure a sufficient number of events in the shortest possible time, drug companies and investigators included most patients with experience of a previous CV event and/or with established CVD.

**Table 1:** Separation of T2D patients participating in CVOTs according to the presence (secondary prevention) or absence (primary prevention) of established CVD

Trial	Drug	Primary prevention	Secondary prevention
EMPA-REG	Empagliflozin	56 (0.8%)	6964 (99.2%)
CANVAS	Canagliflozin	2819 (27.8%)	7323 (72.2%)
DECLARE	Dapagliflozin	10 193 (59.4%)	6967 (40.6%)
VERTIS-CV	Ertugliflozin	82 (1%)	8155 (99%)
SCORED	Sotagliflozin	5440 (51.4%)	5144 (48.6%)
Total		18 590 (35.0%)	34 553 (65.0%)

Table 2 shows the cardiorenal effects of SGLT-2i in primary and secondary prevention. Both EMPA-REG and VERTIS-CV trials included patients in secondary prevention only, and therefore an estimate of their effect in primary prevention of T2D is at present not possible. All five trials are consistent with a similar effect of the five SGLT-2i on HF, both in primary and secondary prevention, pointing to an important therapeutic role in this condition, whatever its genesis.

**Table 2:** Cardiorenal effects of SGLT-2i in primary and secondary prevention in T2D

SGLT-2 inhibitors	EMPA-REG^a^	CANVAS	DECLARE	VERTIS-CV^a^	SCORED
MACE					
Primary	–	Neutral	Neutral	–	Reduced
Secondary	Reduced	Reduced	Neutral	Neutral	Reduced
Heart failure					
Primary	–	Reduced	Reduced	–	Reduced
Secondary	Reduced	Reduced	Reduced	Reduced	Reduced
DKD					
Primary	–	Reduced	Reduced	–	Neutral
Secondary	Reduced	Reduced	Reduced	Neutral	Neutral

^a^Included T2D patients in secondary prevention only

## What’s the road ahead?

Despite treatment for T2D changed markedly in the last two decades, including the use of new medications and combined therapies, overall glycemic control has not improved as much as expected [65]. Among the SGLT-2i approved in the US and in Europe, dapagliflozin has been approved for the treatment of HF, canagliflozin to reduce the risk of ESKD and worsening kidney function in adults with DKD. It is anticipated that novel indications may be issued in the near future as the results of ongoing trials become available. The accumulated evidence suggests that SGLT-2i should be considered for control of hyperglycemia in patients with T2D, particularly in those with established CVD, given that they reduce the risk of MACE, hospitalization for HF and progression of DKD, regardless of whether patients are receiving or not metformin [66].

The remarkable reduction in risk of HF seen across CVOTs, as well as other intervention trials (Fig. 2) and observational data [67] is one major benefits of therapy with SGLT-2i in patients with T2D. Accordingly, the last updated clinical recommendations by ADA exhort clinicians to consider the use of SGLT-2i in patients with T2D and established HF with reduced ejection fraction to reduce the risk of worsening HF and CV death [68]. SGLT-2i have already been included in the first-level prevention guidelines of the 2019 American College Cardiology/American Heart Association for cardiovascular disease [69]. Moreover, evidence from completed trials also indicates that patients with T2D and with lower levels of eGFR and higher levels of albuminuria are among those who stand to gain the greatest absolute benefits [70].

The results of post-hoc analyses [58, 59] or real-world databases [71] are reassuring about both the efficacy and safety of SGLT-2i in older patients with T2D, for whom the choice of the optimal drug is often limited by subjective (health state of patient) and objective (side effects of the antihyperglycemic drug) considerations. SGLT-2is can be used as an add-on therapy in selected and healthy older patients with T2D, especially those who are overweight and have uncontrolled hypertension. For instance, in a multinational observational study [72] including 1,006,577 new users of SGLT-2i or dipeptidyl peptidase-4 inhibitors (DPP-4i), the use of SGLT-2i was associated with a lower risk of HF and CKD versus DPP-4i in patients with T2D otherwise free from both cardiovascular and renal disease.

More research is needed to better delineate the main mechanisms involved in the cardiorenal protection of SGLT-2i (for example focusing more on the cell types target of SGLT-2 inhibition), in order to explain some differences observed in CVOTs. Hopefully, this may lead to new treatment targets in patients with CV risk factors.

## Balancing benefits and harms

The demonstration of cardiorenal benefits in patients with T2D has been the main breakthrough emerged from SGLT-2i trials and observational studies worldwide in the last five years. However, SGLT-2i have also been associated with a variety of adverse effects [73], inducing both the FDA and EMA to publish specific warnings regarding an increased risk of lower-limb amputation, diabetic ketoacidosis, bone fracture, acute renal injury and Fournier gangrene. In the SCORED trial [33], diarrhea, genital mycotic infections, volume depletion, and diabetic ketoacidosis occurred more with sotagliflozin than placebo. Therefore, these medications should be considered with particular attention, and probably better avoided in frail diabetic patients, especially if treated with loop diuretics (due to increased risk of dehydration and orthostatic hypotension) [74], or those with previous limb ulcers or history of amputation. However, a recent meta-analysis [75] of six trials (EMPA-REG, CANVAS, CREDENCE, DECLARE, DAPA-HF, VERTIS-CV) show that SGLT-2i were not significantly associated with an increased risk of amputation, including subgroups with or without established peripheral artery disease. Moreover, the results of a large population-based cohort study [76] indicate that use of SGLT-2i is not associated with an overall increased risk of breast cancer compared with DPP-4 inhibitors, at least in the short term (median follow-up 2.6 years).

## Conclusions

The cardiorenal benefits demonstrated by SGLT-2i are not only limited to patients with T2D, as a mounting evidence indicates that they can also be extended to patients without T2D who have CVD, HF, or CKD [36–38, 48]. Intuitively, the use of these agents will presumably be extended to other medical figures that cooperate in the management of T2D, including cardiologists and nephrologists, within the frame of a multidisciplinary approach, with the paramount aim to improve glycemic control and reduce the risk of cardiorenal events. It seems important that cardiologists and nephrologists become more knowledgeable about T2D and its management, while diabetologists do the same with cardiac and kidney disease, especially those that are prevalent in T2D. At present, only the robust reduction in the risk of hospitalization for HF should be considered a class effect, i.e. embracing the five components of SGLT-2i (empagliflozin, canagliflozin, dapagliflozin, ertugliflozin and sotagliflozin), as emerged by outcome trials.

It is sad to acknowledge that, despite the continuing accumulation of data regarding the benefits of SGLT-2i which are incorporated in newly updated guidelines, many eligible patients with T2D are still not receiving these agents, depriving them of protection against the progression of avoidable cardiorenal complications [77].

## Data Availability

All data generated or analyzed during this study are included in this published article.

## Abbreviations

SGLT-2i: Sodium–glucose transporter-2 inhibitors
MI: Myocardial infarction
ESKD: End stage kidney disease
HF: Heart failure
T2D: Type 2 diabetes
CVD: Cardiovascular disease
A1C: Hemoglobin A1c
MACE: Major adverse cardiovascular events
CKD: Chronic kidney disease
DKD: Diabetic kidney disease
CVOTs: Cardiovascular outcome trials
EMPAREG-OUTCOME: Empagliflozin cardiovascular outcome event trial in type 2 diabetes mellitus patients-removing excess glucose randomized double-blind controlled
CANVAS: Canagliflozin cardiovascular assessment study
DECLARE: Dapagliflozin effect on cardiovascular events randomized double-blind controlled trial
CREDENCE: Canagliflozin and renal events in diabetes with established nephropathy
eGFR: Estimated glomerular filtration rate
VERTIS-CV: Cardiovascular outcomes with ertugliflozin in type 2 diabetes
SCORED: Sotagliflozin in patients with diabetes and chronic kidney disease
DAPA-HF: Dapagliflozin in patients with heart failure and reduced ejection fraction
EMPEROR-R: Cardiovascular and renal outcomes with empagliflozin in heart failure
SOLOIST-WHF: Sotagliflozin in patients with diabetes and recent worsening heart failure
DAPA-CKD: Dapagliflozin in patients with chronic kidney disease
DPP-4i: Dipeptidyl peptidase-4 inhibitors
